# Let-7i enhances anti-tumour immunity and suppresses ovarian tumour growth

**DOI:** 10.1007/s00262-024-03674-w

**Published:** 2024-03-30

**Authors:** Andrew N. Wilkinson, Rui Chen, Elaina Coleborn, Trent Neilson, Khang Le, Chintan Bhavsar, Yue Wang, Sharat Atluri, Gowri Irgam, Kiefer Wong, Da Yang, Raymond Steptoe, Sherry Y. Wu

**Affiliations:** 1 School of Biomedical Sciences, The University of Queensland, Brisbane, QLD 4072 Australia; 2 Department of Pharmaceutical Sciences, University of Pittsburgh, Pittsburgh, PA 15261 USA; 3 Frazer Institute, University of Queensland, Brisbane, QLD 4102 Australia

**Keywords:** miRNA, Let-7i, Cancer immunity, Ovarian cancer

## Abstract

Cancer immunotherapy has seen significant success in the last decade for cancer management by enhancing endogenous cancer immunity. However, immunotherapies developed thus far have seen limited success in the majority of high-grade serous carcinoma (HGSC) ovarian cancer patients. This is largely due to the highly immunosuppressive tumour microenvironment of HGSC and late-stage identification. Thus, novel treatment interventions are needed to overcome this immunosuppression and complement existing immunotherapies. Here, we have identified through analysis of > 600 human HGSC tumours a critical role for Let-7i in modulating the tumoural immune network. Tumoural expression of Let-7i had high positive correlation with anti-cancer immune signatures in HGSC patients. Confirming this role, enforced Let-7i expression in murine HGSC tumours resulted in a significant decrease in tumour burden with a significant increase in tumour T cell numbers in tumours. In concert with the improved tumoural immunity, Let-7i treatment also significantly increased CD86 expression in antigen presenting cells (APCs) in the draining lymph nodes, indicating enhanced APC activity. Collectively, our findings highlight an important role of Let-7i in anti-tumour immunity and its potential use for inducing an anti-tumour effect in HGSC.

**Supplementary Information:**

The online version contains supplementary material available at 10.1007/s00262-024-03674-w.

## Introduction

High-grade serous carcinoma (HGSC) is the most fatal gynaecological cancer with a 5 year survival rate of 47.4% and a median survival time of 52 months [1–3]. Currently, the standard treatment for advanced-stage ovarian cancer is primary cytoreductive surgery and a platinum-based adjuvant chemotherapy [4, 5]. Cancer recurrence is frequent after initial treatment, which leads to poor survival outcomes [6, 7]. Immune therapy represents a promising strategy to improve patient survival by utilising the body’s immune system to eliminate cancer cells [8]. Current immunotherapies for solid tumours include immune checkpoint blockers (ICBs), cancer vaccines, and adoptive cell therapy, with many new therapies currently in early phase trials [9–11]**.** In particular, several checkpoint inhibitors such as anti-PD-L1 (e.g. avelumab), anti-PD-1 (e.g. pembrolizumab, nivolumab), and anti-CTLA-4 (e.g. ipilimumab) have been tested clinically and have shown significant benefits for many cancers. However, only a 9.7–15% objective response rate has been observed in HGSC (NCT01772004, NCT02054806) [6, 12–15]. This low response rate has largely been attributed to the highly immunosuppressive nature of the tumour microenvironment (TME) and low level of T lymphocyte infiltration found in HGSC [16, 17].

The presence of functional cytotoxic T lymphocytes in tumours dictates tumour responsiveness to immunotherapies [18, 19], and additionally tumour-infiltrating lymphocytes (TILs) are a known independent predictor of improved clinical outcomes for ovarian cancer [9, 20]. However, approximately 70% of ovarian tumours lack a sufficient number of functional TILs to combat tumour growth due to the immune suppressive nature of the TME [6, 7, 7, 9, 20, 21]. In ovarian cancer, many immune suppression mechanisms have been identified: CD8^+^ T cell suppression by regulatory T cells (T_regs_), IL-10 and IL-6 mediated upregulation of inhibitory receptor PD-1 on tumour infiltrating CD8^+^ T cells, as well as the presence of immunosuppressive myeloid-derived suppressor cells (MDSCs), tumour-associated macrophages (TAMs), and cancer-associated fibroblasts [6, 22–24]. These result in a decrease in CD8^+^ T cell infiltration and function in ovarian tumours that inhibits the efficacy of immunotherapies. Therefore, a lack of functional TILs is a critical deficit in the required cancer immunity cycle [25] and thus remains a prime target for novel immunotherapies to complement ICB therapies. Strategies being investigated to overcome barriers to CD8^+^ T cell infiltration include targeting molecules expressed by tumour cells, such as CDK4/6, CXCL13, and PD-L2 [26–28] where encouraging results are observed, and further trials have been recommended. While promising, the targeting of individual molecules potentially allows evasion of treatment via pathway redundancy where other immunosuppressive pathways in the TME can compensate for the functions of targeted molecules [11, 29]. A novel treatment to overcome system redundancy is the use of microRNAs (miRNAs), as these endogenous molecules can simultaneously target multiple genes to regulate multiple targets [30].

miRNAs are non-coding, single-stranded RNAs that are 21–23 nucleotides in length [31, 32]. They are partially complementary to the 3’-end of the untranslated region (UTR) of mRNA and can recruit the RNA-induced silencing complex (RISC) to induce translation suppression, degradation, decapping or deadenylation of target mRNA [31, 32]. Use of miRNA for cancer therapy is an emerging field with several drugs including targomiRs, MRG-201, MRG-106, and RG012 currently in clinical testing [33]. Immune processes such as the differentiation and activation of tumour-associated immune cells including macrophages (e.g. miR19a-3p), natural killer (NK) cells (e.g. miR-181), and T cells (e.g. miR-29a-30 and miR-21-5p) are dependent on the expression of certain miRNAs [34–37]. Within tumour cells, miRNAs can regulate antigen processing and presentation by targeting one or more components of the antigen processing machinery and MHC-I molecules [38, 39]. Previous research has shown the ability of miR-326 and miR-340 for enhancing T cell infiltration in lung adenocarcinoma and large B cell lymphoma, respectively [40, 41]. Specifically in ovarian cancer cells, miR-20a and miR-92 have been shown to impact MICA/B and PD-L1 expression, respectively, to affect NK and T cell activity [42, 43]. Furthermore, miR-199a negatively regulates IKKβ mRNA in epithelial ovarian cancer cells, which is needed to induce the NF-κB pathway to secrete pro-inflammatory and pro-tumour cytokines [35]. Although these findings aid understanding of how tumoural miRNA expression is implicated in carcinogenesis, there is currently a lack of comprehensive systematic approaches which directly identify miRNAs important for T cell infiltration and anti-tumour immunity in ovarian tumours. In this study, we performed a systematic analysis integrating patient data and in vitro and in vivo experiments to identify miRNAs important for anti-tumour immunity in HGSC. Let-7i was identified to be an important mediator for this process, and its impact on tumoural immune network is examined in this study.

## Methods

### The cancer genome atlas (TCGA) analysis

LinkedOmics was used to identify miRNAs important for regulating anti-tumour immunity in HGSC [44]. The platform was developed using TCGA dataset and contains tumour genomic information from 602 ovarian serous adenocarcinoma patients. First, the symbols of all miRNAs in the human genome were collected. This was done by developing a Python script to parse all gene symbols beginning with ‘MIR’ from NCBI Gene Info file (https://www.ncbi.nlm.nih.gov/gene/), 2002 symbols were found. All 2002 miRNAs were then entered into LinkedOmics, each one being subjected to the following search options: Cancer Cohort: TCGA_OV, Search Dataset: miRNA Seq, Target Dataset: RNAseq, Statistical Method: Pearson Correlation. Of the 2002 genes entered into LinkedOmics, 521 were found to have data for miRNA Sequencing in the Ovarian TCGA database. The LinkedInterpreter module of LinkedOmics was subsequently used to perform the enrichment analysis with the following parameters: Tool: Gene Set Enrichment Analysis (GSEA), Rank Criteria: FDR, Minimum Number of Genes: 3, Simulations: 500.

miRNAs were then prioritised based on immune-related GO terms. These terms were determined by gathering the list of all GO terms and their descriptions and searching for immunological-related words of interest using Python. There were 3877 immunological-related terms out of all 51,281 GO terms. The filtered data were outputted to csv file, and each significant immune-related GO term was counted for each miRNA and presented using Pandas Python. The data were plotted using Seaborn and Matplotlib bar, heatmap and custom scatter (bubble) plot modules. Codes for plots are available at this link: https://github.com/secretx51/Let7i-Data-Figure-Generation.

For correlation with anti-cancer immune signature analysis, enrichment score of 68 immune signatures previously reported [45] was calculated in single-sample gene set enrichment analysis (ssGSEA). Clinically annotated data from TCGA obtained from the Open-Access and Controlled-Access tiers of the TCGA Data Portal (http://tcga-data.nci.nih.gov/tcga/findArchives.htm) were used with NIH approval. Total of 347 HGSC patients were included in the analysis. miRNA expression data were obtained from Agilent miRNA microarrays and Illumina miRNA-Seq data sets. For the miRNA-Seq data, we derived the ‘reads_per_million_miRNA_mapped’ values for mature forms for the miRNA examined from the ‘isoform_quantification’ files. The correlation analyses were carried out by Python (version 3.8.0) (http://www.python.org/).

### Cell culture

ID8 murine HGSC cells were generated and kindly provided by Prof Roby from University of Kansas, and ID8-ip1-Luc cells were generated from isolation of tumour cells after ID8 tumour engraftment in a female C57BL/6 mouse followed by luciferase labelling. Cells were grown in high-glucose Dulbecco's Modified Eagle's Medium (DMEM, Sigma-Aldrich) supplemented with 7% foetal bovine serum (FBS, Sigma-Aldrich), insulin–transferrin–selenium (1X ITS, Lonza), and 1% penicillin–streptomycin (Sigma-Aldrich). Cells were authenticated to ensure no cross contamination with other cell lines, and all cells were tested negative for contamination with *Mycoplasma.*

### Generation of let-7i-expressing cell lines

ID8-ip1-Luc-miR-Ctrl and ID8-ip1-Luc-let-7i stable expressing cell lines were generated by transducing ID8-ip1-Luc cells using SMARTvector Non-Targeting mEF1a-TurboGFP and SMARTvector let-7i-5p mEF1a-TurboGFP vectors (Dharmacon), respectively. After transduction, cells were cultured in growth medium containing puromycin for selection. Expression of Let-7i in these transduced lines was assessed using Taqman miRNA quantitative PCR.

### Nanoparticle preparation

miRNA-containing liposomal formulations were prepared as previously described [46, 47], using the hydration of freeze-dried matrix method. Dioleoyl trimethylammonium propane (DOTAP, 18:1), cholesterol, and polyethylene glycol (PEG)2000-C_16_Ceramide were purchased from Sigma. For all formulations, a nitrogen/phosphate (N/P) ratio of 4:1 was used, and formulation were designed to reach a 20 µg of miRNA per 200 uL volume concentration once hydrated. miRNA was diluted in sucrose solution (0.925 mg of sucrose used per 1 µg of miRNA) and mixed with equal volume of cholesterol and PEG2000-C_16_Ceramide dissolved in tert-butanol. This formulation was snap-frozen then freeze-dried overnight (BenchTop Pro, Omnitronics) at a condensing temperature of − 80 °C and pressure of less than 0.1 mbar. The lyophilised product was dissolved in nuclease-free water with gentle shaking and sonication prior to injection. When hydrated at 20 µg miRNA/200 µL concentration, the sucrose in solution makes the formulation isotonic and ready for in vivo use.

### Mice

All mice experiments were approved by University of Queensland (UQ) Animal Ethics Committee. Female C57BL/6 J mice (6–8 week old) were purchased from ARC and housed in UQ Centre of Advanced Imaging animal facility. Luciferase-labelled ID8-ip1 cells, ID8-ip1-miR-Ctrl, or ID8-ip1-Let-7i (1.5 × 10^6^ cells/mouse) were implanted into mice via intraperitoneal (i.p.) injection. Tumour growth and establishment were monitored every week via i.p. injection of luciferin and bioluminescence imaging was performed 7–9 min post-injection. Luciferin bioluminescence images were acquired using IVIS Lumina X5 imaging system and analysed using in vivo imaging software. For the nanoparticle study, mice received PEGylated DOTAP NPs containing either negative control miRNA (miR-Ctrl) or Let-7i intravenously given twice weekly (20 µg/dose)[46] starting from day 6 post tumour inoculation. Mice received 3 weeks of treatment. For all mice experiments, ascites fluid was collected from mice at experimental endpoint. Tumours in omentum and other organ sites alongside inguinal and mesenteric lymph nodes (LNs) were dissected from mice in a double-blinded manner. Tissues were kept in FACS buffer (2% FBS and 5 mM EDTA in PBS) for flow cytometry analysis or snap frozen in liquid nitrogen for RNA analysis.

### Flow cytometry

Omental tumour and LN tissues were mashed through a 70 µm cell strainer to acquire single cell suspension. Cells were centrifuged at 500 rcf for 5 min at 4 °C, washed with FACS buffer twice, then resuspended in 85 µL FACS buffer. Cells were then incubated with anti-mouse CD16/CD32 monoclonal antibody (1:200, BD Biosciences, Cat# 553142) for 15 min at 4 °C. Antibodies or respective isotype controls listed in Supplementary Table 1 were diluted in FACS buffer and used to surface stain cells for 20 min at 4 °C. Precision Count Beads (Biolegend) were additionally added to allow quantification of the total number of immune cells in each sample. BD Fortessa X-20 flow cytometer and FlowJo software were used to analyse samples. Immune cell populations were defined as listed in Supplementary Table 2.

### Cell growth assessment in vitro

Growth of ID8-ip1-Luc-Let-7i and ID8-ip1-Luc-miR-Ctrl cells was monitored by seeding 5000 cells/well in a 6 well plate and counting the total number of cells for 5 consecutive days.

### RNA extraction, cDNA synthesis, and miRNA quantitation

RNA was extracted from cells once they reached 80–90% confluency using TRIzol reagent (Life Technologies) according to manufacturer’s protocol. NanoDrop-One (Thermo-Fisher) was used to quantify both RNA quality and concentration. For detection of Let-7i for in vitro cell lines, 100 ng of RNA was reverse-transcribed using TaqMan MicroRNA Reverse Transcription Kit (Thermo-Fisher, Cat# 4366596) and quantified using TaqMan MicroRNA Assay Kit (Thermo-Fisher, Cat# 4427975) according to manufacturer’s protocol. The 2^−ΔΔCt^ method was used to calculate the relative quantity of Let-7i present in each sample, with the expression of SNO135 used to normalize data. For absolute quantitation of microRNAs, 4 ng of RNA was reverse-transcribed using TaqMan MicroRNA Reverse Transcription Kit followed by quantitation using the QIAcuity digital PCR system (Qiagen) according to manufacturer’s protocol. Results were analysed using the QIAcuity software suite.

### Statistical analysis

Correlation analyses was performed using Pearson Correlation test in LinkedOmics platform. Spearman’s correlation was used to assess miRNA/gene signature correlation using TCGA dataset. In vitro and in vivo experimental data analysis was conducted with GraphPad Prism version 8 software. Unpaired two-tailed Student’s t-test was used for statistical analysis for in vitro and in vivo experiments. Two-way ANOVA with Sidak’s multiple comparisons test was used when assessing impact of the treatment on different immune cell populations. Statistical significance was defined by a *p* value < 0.05. Standard error of the mean is shown in all figures.

## Results

### Let-7i positively correlated with immune activity in human ovarian tumours

To determine which miRNAs have highly positive correlation with anti-tumour immunity in HGSC, enrichment analysis based on Gene Ontology (GO) was first performed on the TCGA database using LinkedOmics platform [44]. LinkedOmics platform was chosen specifically because of its high efficacy in determining the downstream pathways of miRNAs using human samples. The LinkInterpreter module from LinkedOmics transforms and identifies associations into biological understanding, through pathway and network analysis. The module was set to perform the analysis searching from the Ovarian cancer miRNAseq dataset and uses bulk RNA seq data. This was done for all 521 miRNAs in the ovarian miRNAseq TCGA dataset. The data were then filtered to include only GO terms relevant for immune or cytotoxic T cell functions, which was determined to be 3877 of the total 51,821 GO terms. The miRNAs were then ranked based on the number of significant immunological pathways identified (*p* < 0.05).

**Fig. 1:**
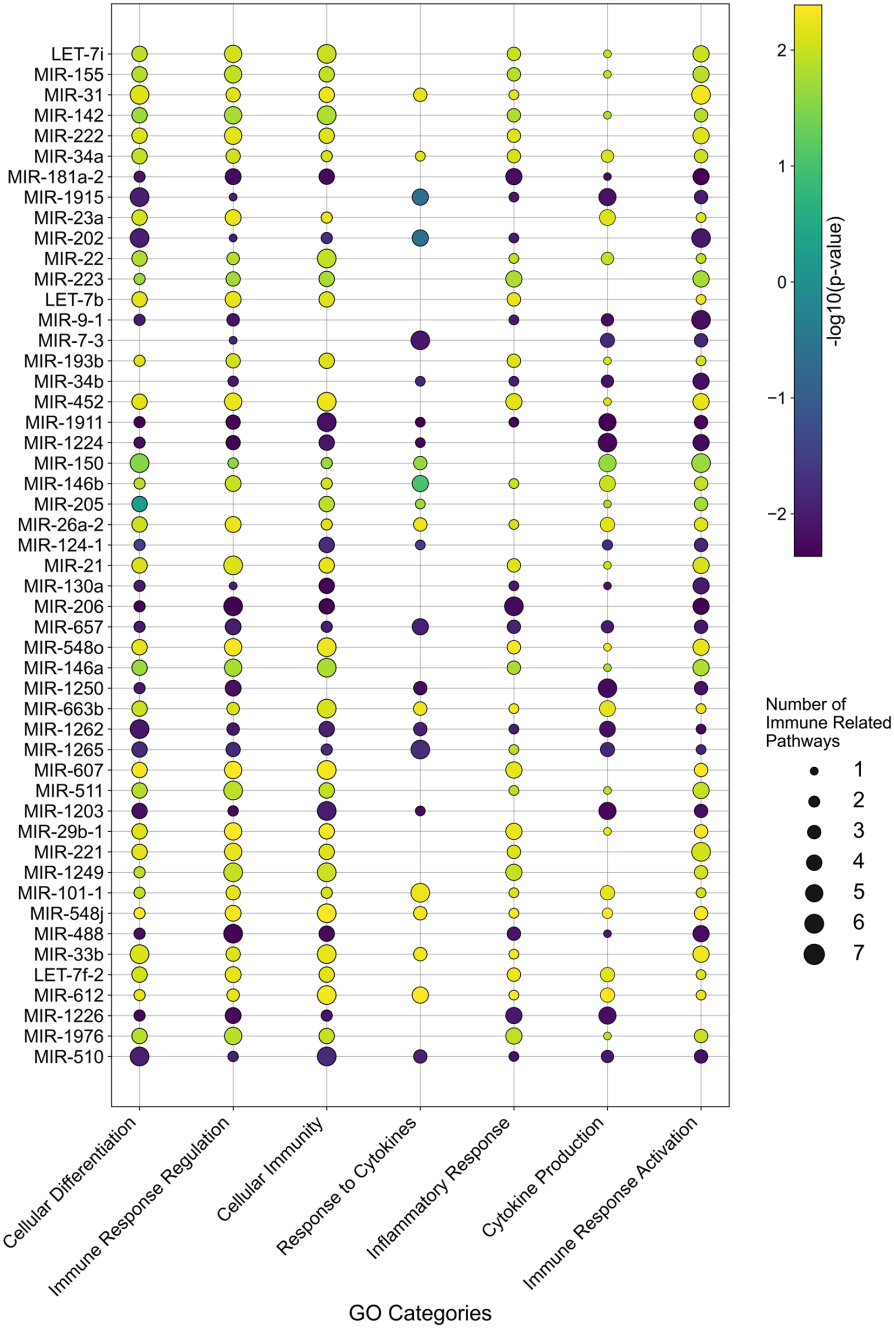
Correlation of miRNA expression in HGSC immunological pathways. miRNAs are ranked according to the number of impacted immunological pathways (*p* < 0.05), depicted across categories by the size of the bubbles. The significance of the correlations is indicated by the colour of the bubbles shown (-Log_2_ *P* Value). Spearman’s correlation analysis was performed

**Fig. 2:**
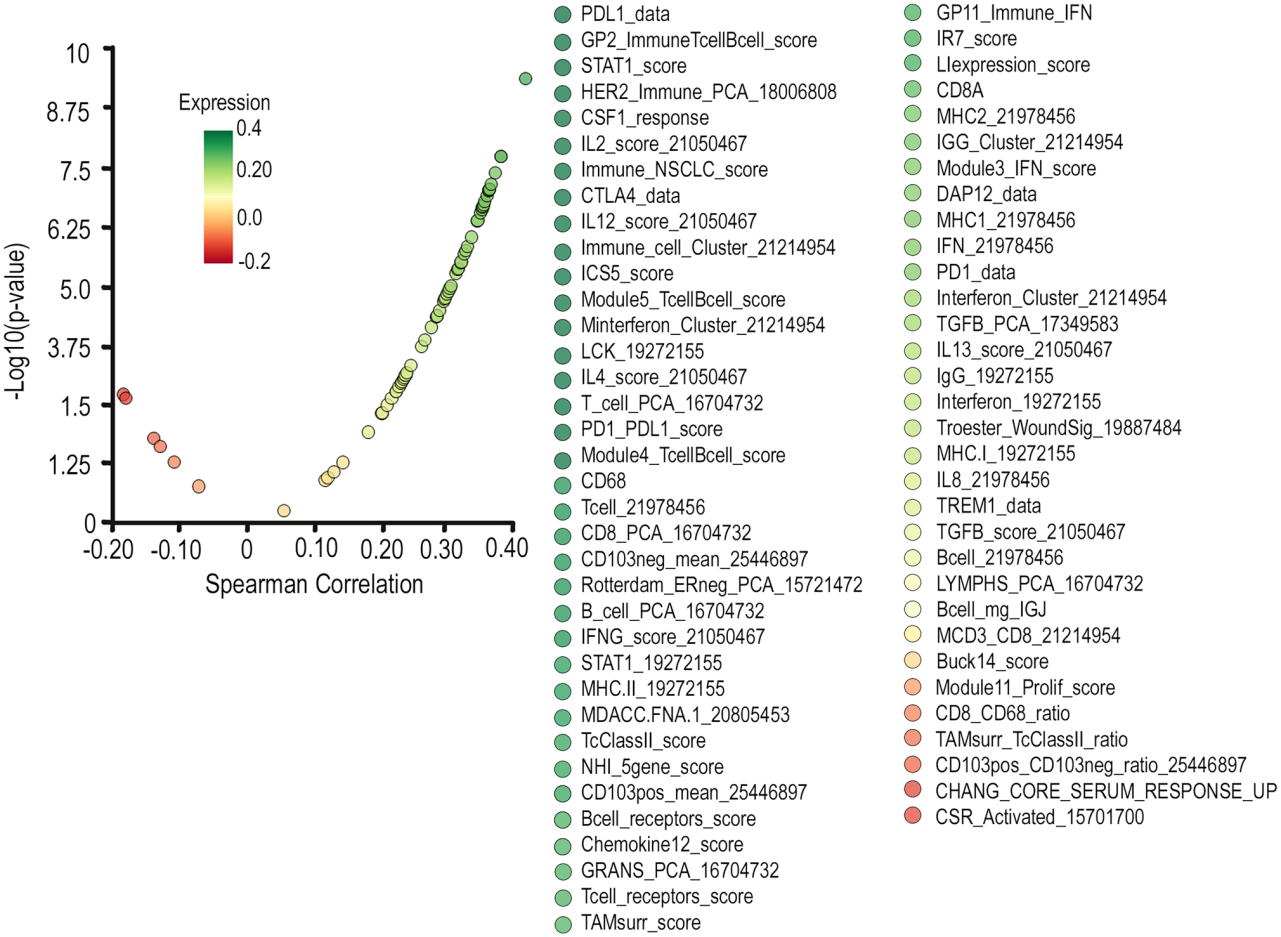
Correlation between tumoural Let-7i expression and anti-cancer immune signatures in TCGA ovarian cancer dataset. Spearman’s correlation was used to assess miRNA/gene signature correlation using TCGA dataset (*n* = 347)

From this analysis, Let-7i was found to impact the greatest number of immunological pathways out of all tested miRNAs in the ovarian miRNAseq dataset (Supplementary Fig. S1). Out of the 7 categories examined (Fig. 1), Let-7i in comparison to other miRNAs had a more significant impact on two categories: immune response regulation and inflammatory response. These two categories included GO pathways related to lymphocyte regulation and antigen processing/presentation, which are highly relevant for generation of effective anti-tumour immune response. GO terms included in all categories are listed in Supplementary Table 3. When looking at the individual GO immunological pathways that Let-7i acts on, it has the greatest significance by log2 *P* Value of all miRNAs for leukocyte and myeloid dendritic cell activation pathways (highlighted in red, Supplementary Fig. S2). This evidence points towards the potential role of Let-7i in regulating immune responses in HGSC. Indeed, in TCGA ovarian cancer dataset, correlation analysis between tumoural Let-7i expression and the 68 anti-cancer immune signatures previously reported [45] revealed that Let-7i had an overall positive correlation with these gene signatures important for anti-cancer immunity (Fig. 2).

### Increased Let-7i expression significantly reduces ovarian tumour growth in vivo

**Fig. 3:**
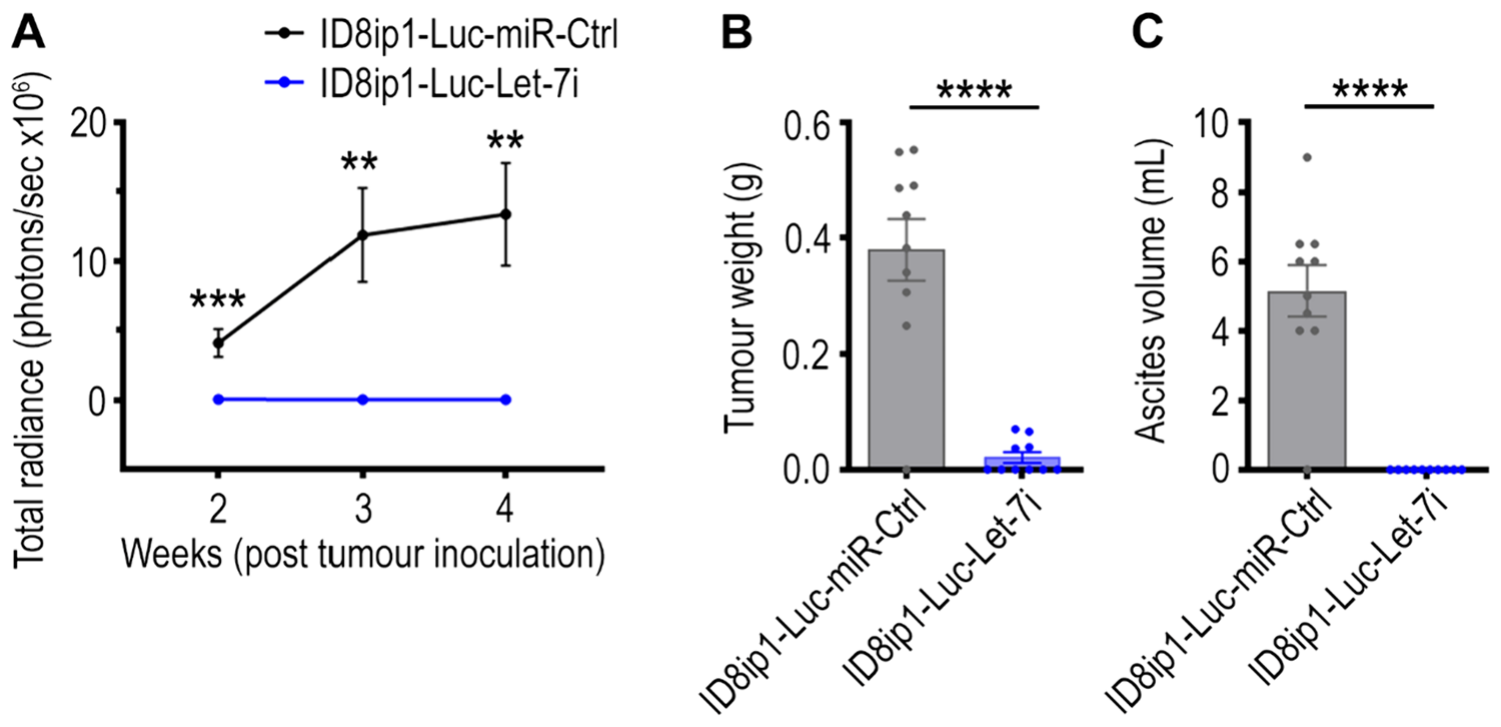
Impact of constant Let-7i expression on tumour burden in mice bearing ID8ip1-Luc-miR-Ctrl or ID8-ip1-Luc-Let-7i tumours. C57BL/6 J mice were i.p injected with 1.5 × 10^6^ luciferase-labelled ID8ip1-Luc-miR-Ctrl or ID8-ip1-Luc-Let-7i cells and mice were sacrificed 28 days post tumour inoculation. **A** Tumour growth in mice indicated by bioluminescence signal throughout the experiment. Total radiance signal (photons/sec) was quantified using IVIS imaging system. **B** Total tumour weight and **C** ascites volume at experiment endpoint. All bars and error bars represent mean ± SEM (**, *p* < 0.01; ***, *p* < 0.001; ****, *p* < 0.0001, *n* = 10/group). Statistical analyses were performed by unpaired Student’s *t* test

Given the impact of Let-7i on ovarian tumour biology has been largely unexplored to date, we first investigated whether tumoural Let-7i expression can reduce ovarian tumour progression in vivo. C57BL/6 J mice were inoculated with transduced murine ID8-ip1 cells that have constant forced Let-7i or miR-Ctrl (control) expression. ID8-ip1 was chosen as the model murine HGSC line for this study as it is a widely used to study ovarian tumour biology in immune competent mouse models. Validation of Let-7i expression in transduced ID8-ip1 cells prior to transplantation showed a 6.9-fold increase in Let-7i in ID8-ip1-Luc-Let-7i cells compared to their ID8-ip1-Luc-miR-Ctrl cells (*p* < 0.0001, Supplementary Fig. S3). Tumour growth was monitored via luminescence imaging of luciferase-tagged ID8-ip1-Luc-miR-Ctrl and ID8-ip1-Luc-Let-7i tumours (Supplementary Fig. S4). A significant decrease in tumour signal were seen within ID8-ip1-Luc-Let-7i bearing mice at weeks 2, 3, and 4 post tumour inoculation (Fig. 3A). Consistently, at endpoint, the mice bearing ID8-ip1-Luc-Let-7i tumours had a significant reduction in total tumour weight by 94.4% compared to mice bearing ID8-ip1-Luc-miR-Ctrl tumours (*p* < 0.0001, Fig. 3B). This effect is also mirrored in ascites volume, where a significant reduction was seen in the ID8-ip1-Luc-Let-7i tumour bearing mice (*p* < 0.0001, Fig. 3C). Altogether, these data indicate that an increase in tumoural Let-7i expression drives an anti-tumour effect.

To assess whether this anti-tumour effect could be caused by Let-7i intrinsically impacting cell growth pathways within the tumour cells, we monitored the growth patterns of the ID8-ip1-Luc-Let-7i and ID8-ip1-Luc-miR-Ctrl cells in vitro. No significant difference was observed for the growth rate between these two transduced cell lines (Supplementary Fig. S5). This suggests that Let-7i does not have a major or significant impact on intrinsic cell growth mechanisms of these cancer cells, but impacts the in vivo tumour microenvironment to reduce tumour growth. We hypothesise that the anti-tumour effect observed following Let-7i treatment is likely due to its regulation of the tumoural immune network given the strong association of Let-7i with immune pathways in human ovarian tumours (Figs. 1 and 2).

### Let-7i enhances T cell tumour infiltrates and activity of antigen presenting cells

**Fig. 4:**
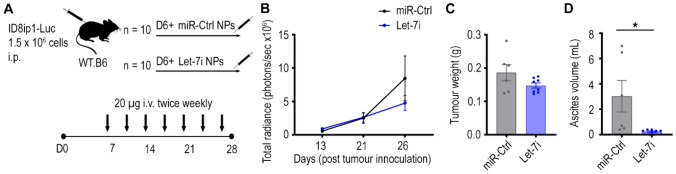
Impact of Let-7i NP treatment on tumour burden in ID8-ip1-Luc tumour model. **A** C57Bl/6 J mice were i.p. injected with 1.5 × 10^6^ luciferase labelled ID8-ip1-Luc cells and treated with i.v. injection of NPs containing either non-targeting miRNA control (miR-Ctrl) or Let-7i, at 20 µg/dose. NP treatment started at 6 days post tumour inoculation and doses were given twice per week for 3 weeks. Ascites was drained and measured, and tumours in omentum (primary site of tumour growth) as well as tumours growing in other parts of the peritoneal cavity were dissected. **B** Tumour growth was monitored throughout the duration of the experiment by bioluminescence imaging of luciferase signal. Total radiance signal (photons/sec) was quantified using IVIS imaging system. **C** Total tumour weight of mice at experiment endpoint. **D** Volume of ascites in mice at endpoint. All bars and error bars represent mean ± SEM (*, *p* < 0.05; miR-Ctrl group, *n* = 6; Let-7i group, *n* = 8). Tumours did not develop for four and two mice for miR-Ctrl and Let-7i treatment groups, respectively. Statistical analyses were performed by unpaired Student’s *t* test

**Fig. 5:**
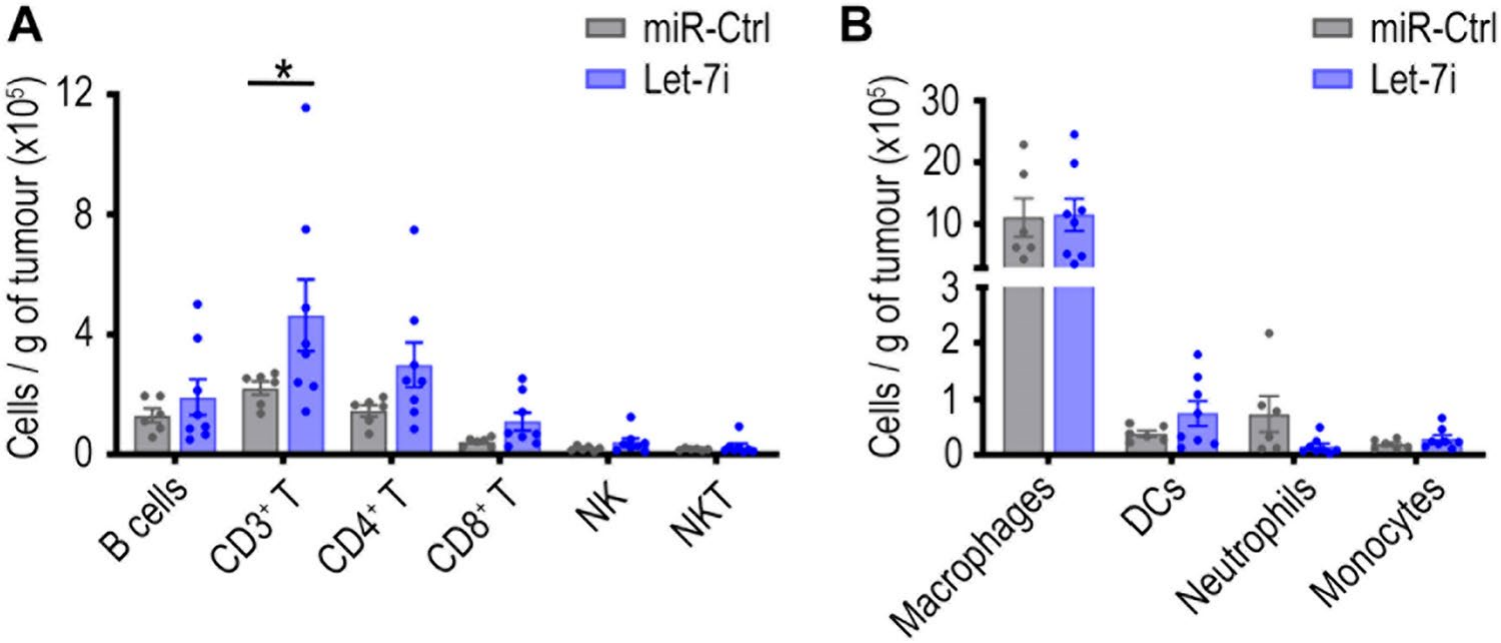
Impact of Let-7i NP treatment on immune cell populations in ID8-ip1-Luc tumour model. Omental tumours from ID8-ip1 tumour bearing mice treated with Let-7i or miR-Ctrl NPs were profiled by flow cytometry to examine the frequencies of **A** lymphoid and **B** myeloid immune cells per gram of tumour at experiment endpoint. All bars and error bars represent mean ± SEM (*, *p* < 0.05; miR-Ctrl group, *n* = 6; Let-7i group, *n* = 8). Statistical analyses were performed by two-way ANOVA with Sidak’s multiple comparisons test

We next investigated the impact of Let-7i on immune cell networks within tumours. Given that the ID8-ip1-Luc-Let-7i model had tumours of extremely low weight, we conducted an additional experiment which would procure tumours of adequate sizes for such analysis and also more closely resemble how Let-7i could be therapeutically delivered to tumours in human patients. For this experiment, we inoculated C57BL/6 J mice with ID8-ip1 cells and after allowing tumours to establish for 1 week, we intravenously injected mice with nanoparticles (NPs) containing Let-7i or a non-targeting negative control miRNA (miR-Ctrl) (Fig. 4A). These NPs take advantage of the enhanced permeability and retention effects in solid tumours to passively target tumours [46]. The experiment was terminated at 28 days post tumour inoculation, as this was when luminescence imaging of tumours suggested Let-7i NP treated tumours began to have an impact on tumour growth rate compared to the control group and sizeable tumours were needed for the immunological analyses (Fig. 4B**, **Supplementary Fig. S6). Consistent with luminescence imaging results, average tumour weight in Let-7i NP treatment group was slightly reduced compared to miR-Ctrl-treated tumours but still of sufficient size for detailed immune assessment (Fig. 4C). Ascites volume was notably reduced in mice treated with Let-7i NPs compared to control (Fig. 4D). Let-7i expression was indeed higher in the Let-7i NPs treatment group indicating successful delivery of Let-7i mimics using the nanoparticles (Supplementary Fig. S7A). While this level of Let-7i increase is much lower than what was observed in transduced cell lines (Supplementary Fig. S3), absolute quantification by digital PCR indicates that this level of increase corresponds to an average of 5487,118 copies of Let-7i being delivered to each tumour (Supplementary Fig. S7B). Full immune profiling was performed in tumours obtained from the omentum, a major ovarian cancer metastatic site in human patients and in ID8-ip1 mouse model, immediately after dissection. For lymphoid populations, there was a trend of an increase in number of tumour infiltrating lymphocytes (CD3^+^ T, CD4^+^ T, CD8^+^ T, and B cells) per gram of tumour (Fig. 5A, Supplementary Fig. S8A), although the CD8^+^ T cells had a comparable frequency of memory cells in both treatment groups (Supplementary Fig. S9). These trends were also observed when considering the percentage of these cells out of all CD45^+^ cells (Supplementary Fig. S10A). Minimal changes were seen in the number of NK and NKT cells in tumours following Let-7i treatment (Fig. 5A, Supplementary Fig. S10A).

For myeloid populations, a strong trend of decrease in number of neutrophils per gram of tumour was observed in Let-7i NP treated mice (Fig. 5B, Supplementary Fig. S8B), with this trend also seen in neutrophil portions out of all CD45^+^ leukocytes (Supplementary Fig. S10B). As tumour-associated neutrophils are considered part of the MDSCs population and often have immunosuppressive qualities [48], this indicates Let-7i delivery to tumour could reverse immunosuppression in these tumours. Minimal changes in macrophage numbers were observed. For DCs, a trend of increase in cell numbers per gram of tumour was seen in mice which received Let-7i NP treatment (Fig. 5B). This observation was seen however to a lesser extent when considering their percentage out of CD45^+^ cells within tumour (Supplementary Fig. S10B).

**Fig. 6:**
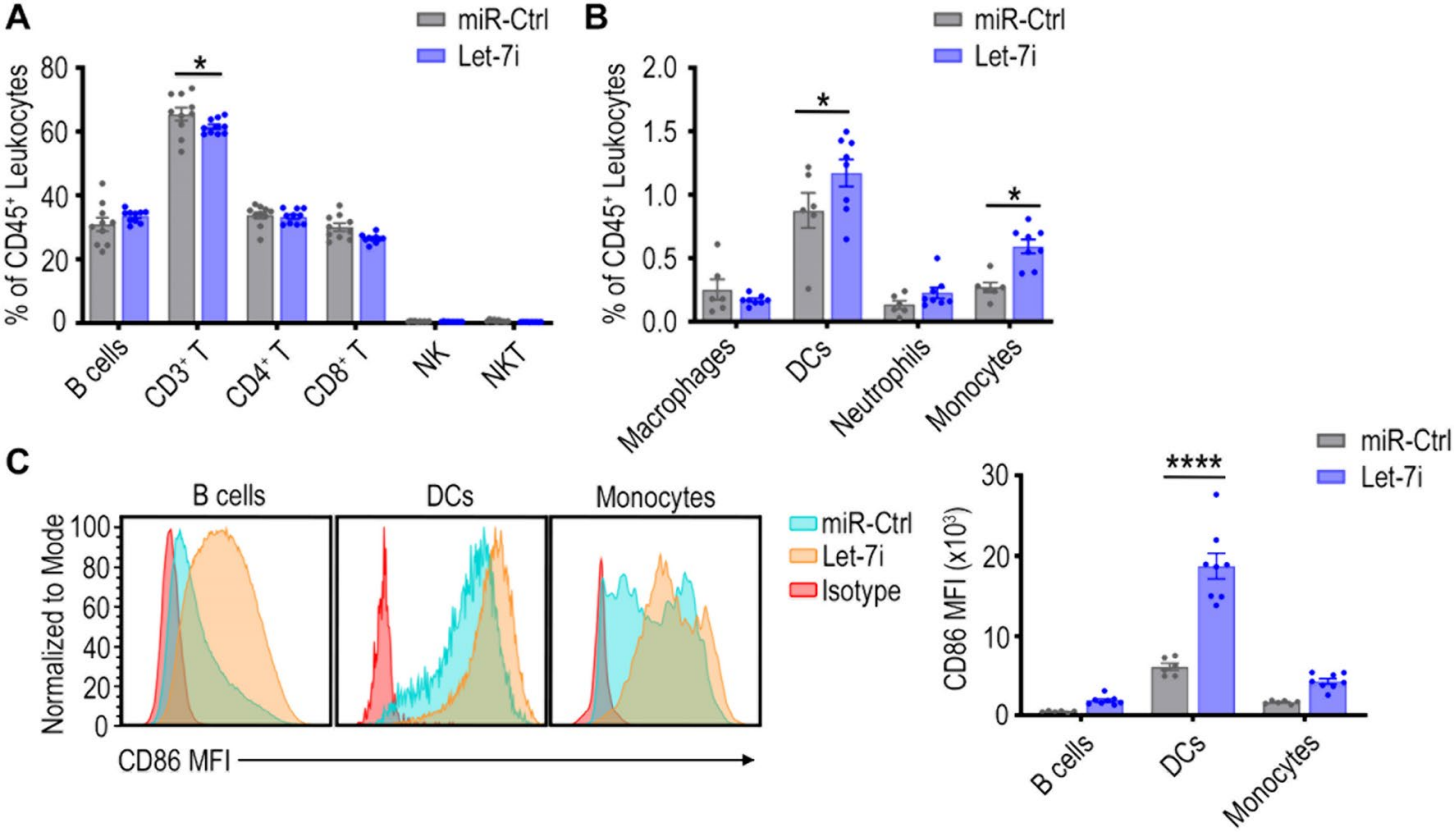
Impact of Let-7i NP treatment on immune cell populations in lymph nodes of ID8-ip1-Luc tumour bearing mice. Inguinal and mesenteric lymph nodes (LNs) from ID8-ip1-Luc tumour bearing mice treated with Let-7i or miR-Ctrl NPs were profiled by flow cytometry to examine the percentage of **A** lymphoid and **B** myeloid immune cells out of all CD45^+^ leukocytes within LNs at experiment endpoint. **C** CD86 mean fluorescence intensity (MFI), a co-stimulatory receptor and activation marker, was assessed on antigen presenting cells; B cells, DCs, and monocytes in LNs as a representative histogram (concatenated samples per group) and bar graph. All bars and error bars represent mean ± SEM (*, *p* < 0.05; **, *p* < 0.01; ****, *p* < 0.0001; miR-Ctrl group, *n* = 6; Let-7i group, *n* = 8). Statistical analyses were performed by two-way ANOVA with Sidak’s multiple comparisons test

As we had observed trends of increase in APC numbers (e.g. B cells, DCs) in tumours after Let-7i NP delivery, we assessed whether the activity of these APCs were also impacted by Let-7i treatment. Within the draining lymph nodes, the usual site of T cell co-stimulation by APCs, some minor changes in the number of T cells, DCs, and monocytes were observed with Let-7i NP treatment (Fig. 6A–B, Supplementary Fig. S11); however, most notably, a significant change in the CD86 expression on DCs was observed with treatment of Let-7i (Fig. 6C). Overall, the mean fluorescent intensity (MFI) of CD86 was increased by 3.33, 3.03, and 2.47-fold for B cells, DCs, and monocytes, respectively, in the Let-7i treatment group compared to the control group. CD86 is a co-stimulatory marker on APC that is upregulated upon activation that in turn activates T cells through co-stimulation of CD28 [49, 50] and therefore indicates an enhanced ability to induce T cell immunity. Within tumours, there was also a trend of an increase in CD86 MFI in APCs (Supplementary Fig. S12). The results suggest improved ability of these APCs to activate T cells, which is a crucial step in initiating cancer immunity for an anti-tumour response. Altogether, these in vivo immune data support the major pathways identified to be closely associated with Let-7i expression in human HGSC tumours (Fig. 1, Supplementary Fig. S2).

## Discussion

Despite the promise of immunotherapies such as immune checkpoint inhibitors for treatment of many cancer types, ovarian cancer lacks a significant response rate to ICIs due to the highly immunosuppressive tumour microenvironment [6–9]. miRNAs represent a promising strategy to overcome immunosuppression within the tumour and thus improve treatment outcomes for ovarian cancer patients. Through multiple lines of evidence, including analyses of 602 human HGSC tumours using the LinkedOmics platform, we have identified the critical role that Let-7i plays in modulating tumoural immune network. We found that enhancing Let-7i expression in ovarian tumours significantly decreases tumour burden, increases the activity of APCs in lymph nodes, and increases T cells presence within tumours. The study highlights the potential of utilising Let-7i to enhance anti-tumour immunity in HGSC tumours and represents the first study to systematically characterise the therapeutic and immunological effect of Let-7i in tumours.

The Let-7 family has previously been shown to be important regulators of the immune response in various pathologies including cancer [51, 52]. However, little has been investigated for the specific Let-7i miRNA. The Let-7 family appears to exert both pro- and anti-tumour effects within cancer cells, thus highlighting a potential benefit to dissecting and individually targeting the important members. Members of the Let-7 family can inhibit Fas expression to desensitise cells to Fas-related apoptosis, while also inhibiting immune evasion in head and neck squamous cell carcinoma (HNSCC) via increased degradation of PD-L1 [53–56]**.** Specifically, for Let-7i, previous studies have found that it reduces cancer cell proliferation and migration through downregulating ERK3 expression in head and neck cancer, as well as HGMCA1 expression in bladder cancer [57, 58]. Specifically in ovarian cancer, Let-7i upregulation decreased stemness and self-renewal, reduced anchorage-independent growth, decreased functional phenotypes associated with metastasis and increased sensitivity to PARPi and platinum-based therapies [59, 60]. We are the first to assess the in vivo effects of Let-7i therapeutic delivery in any tumour model to our knowledge and found Let-7i has a significant therapeutic effect on ovarian tumours. Furthermore, our data indicate that Let-7i specifically impacts the tumoural immune networks, rather than intrinsically on cancer cell growth mechanisms, providing another piece of evidence for mechanism of action for Let-7i for ovarian cancer treatments.

Our data are consistent with other studies that have shown a role for the Let-7 family in regulating adaptive immune responses [52]. In activated CD8 T cells, reduced Let-7 g enhances clonal expression and effector function [52]. A recent study demonstrated that Let-7 family can promote memory and antagonise terminal differentiation in CD8 T cells [61]. Other studies have observed that Let-7 family expression affects differentiation of effector CD8 T cells with high Let-7 needed to maintain naïve phenotype [35, 62, 63]. We did not observe any negative impact on CD8 T cells in our study using Let-7i, again highlighting the potential advantage of targeting specific members of the Let-7 family. While Let-7i NP treatment is not expected to directly influence lymphocytes as it has been well established that CD8 T cells do not take up NPs well in vivo [64], it is possible that other types of immune cells may take up these NPs and mediate the immune effects observed in this study. When Let-7i is only introduced to tumour cells, a significant inhibition of tumour growth was observed in vivo (Fig. 3) but not in vitro in the absence of any other immune cells (Fig. S5). Together, these data suggest that Let-7i expression in tumour cells has a significant indirect effect on tumour microenvironment that enhances anti-tumour immunity, although whether the immune effects observed following Let-7i-loaded NP treatment is driven primarily by its impact on tumour cells remains to be further investigated. Nevertheless, the observed trend of increase in T cells in tumours following Let-7i treatment highlights the potential for Let-7i as an immunotherapy as the presence of TILs is significantly associated with improved outcomes and longer overall survival [65–67].

Interestingly, we found that introducing Let-7i to tumours resulted in enhanced CD86 expressions in APCs in the draining LNs, an important costimulatory molecule that activates and differentiates T cells through interaction with CD28 and is associated with improved APC function [49, 50]. These data also further support the correlation between Let-7i miRNA in HGSC and immunological signatures for activation and differentiation (Fig. 1, S2). However, due to the broad impact of Let-7i on the immune system, the exact mechanism remains unclear, including whether the Let-7i-loaded NPs are taken up by the tumour cells, the APCs, or both to induce anti-tumour immunity. Although as NPs used in this study accumulate in the tumour site through passive targeting approach taking advantage of the enhanced permeability phenomenon in tumours [46], our data suggests that Let-7i likely requires a close interaction between the tumour microenvironment and key APCs to induce this anti-tumour immunity. Future studies may focus on further exploring the mechanism of Let-7i-induced immunity by dissecting the roles of the individual cell types in Let-7i-NP uptake and function.

Poor APC function is major hurdle in overcoming immune suppression as mature dendritic cells are essential for activating T cells, presenting tumour neoantigens, and tumour clearance. Supporting this role, studies have found that an increase in APC maturation in the LN causes significant killing of target cancer cells [68, 69]. The effect of Let-7i on APC phenotype is complementary to previously reported impact of other let-7 family members (let-7a, let-7b) in HNSCC tumours where their expression resulted in decreased PD-L1 expression in cancer cells [56]. This would overall contribute to enhanced T cells’ anti-tumour effects. Importantly, compared to all the other Let-7 family, Let-7i had the strongest correlation with tumoural immune pathways in the > 600 human tumours examined in this study, highlighting its utility in enhancing anti-tumour immunity in HGSC. This complements well with the previously reported role of Let-7i in sensitising cancer cells to PARPi and platinum-based therapies in HGSC [59, 60]. Future work should focus on validating Let-7i’s impact on tumour immunity in other murine models of ovarian cancer as well as its ability to generate tumour antigen-specific immune response. Further improvement on the nanoparticle system to deliver Let-7i to tumours is also needed to further potentiate its impact on anti-tumour immunity. The use of Let-7i with other immune therapies should also be further investigated (e.g. other types of immune therapies or other microRNAs). For instance, based on the cellular mechanism of Let-7i described here, Let-7i could combine well with miR-155, which has been shown to regulate MHC-II and co-stimulatory markers in DCs in lymph nodes [70]. The ability of both Let-7i and miR-155 to positively modulate APC function in LNs could result in possible synergistic effect when used in combination to promote T cell priming and tumour clearance. Finally, an increase in functional T cells in the tumour microenvironment is a critical determinate of anti-cancer immunity and is a requirement for an effective response to immune checkpoint blockade. Collectively, our findings highlight the impact of Let-7i on tumoural immune networks and its potential use for inducing an anti-tumour effect in HGSC.

### Supplementary Information

Below is the link to the electronic supplementary material.

Supplementary file 1 (DOCX 5050 kb)

## Data Availability

The data supporting the findings of this study are available from the corresponding author upon request.
