# Supplementary material for: Let-7i enhances anti-tumour immunity and suppresses ovarian tumour growth
Source: Cancer Immunol Immunother. 2024 Mar 30;73(5):80. doi: 10.1007/s00262-024-03674-w (PMC10981620; doi:10.1007/s00262-024-03674-w)
Supplement: Supplementary file 1 — (DOCX 5050 kb) [file 262_2024_3674_MOESM1_ESM.docx]

**Let-7i enhances anti-tumour immunity and suppresses ovarian tumour growth**

Andrew Wilkinson^1^, Rui Chen^1^, Elaina Coleborn^1^, Trent Neilson^1^, Khang Le^1^, Chintan Bhavsar^1^, Yue Wang^2^, Sharat Atluri^1^, Gowri Irgam^1^, Kiefer Wong^1^, Da Yang^2^, Raymond Steptoe^3^, Sherry Y. Wu^1^

Affiliations:

1. School of Biomedical Sciences, The University of Queensland, Brisbane, Queensland Australia
2. Department of Pharmaceutical Sciences, University of Pittsburgh, Pittsburgh, PA, USA
3. Frazer Institute, University of Queensland, Brisbane, Australia.

*Corresponding Author: Sherry Y. Wu

School of Biomedical Sciences,

The University of Queensland

Brisbane, Queensland, 4072, Australia

[sherry.wu@uq.edu.au](mailto:sherry.wu@uq.edu.au)

ORCID ID: 0000-0002-6051-4252

**Supplementary Figures and Figure Legends**

**
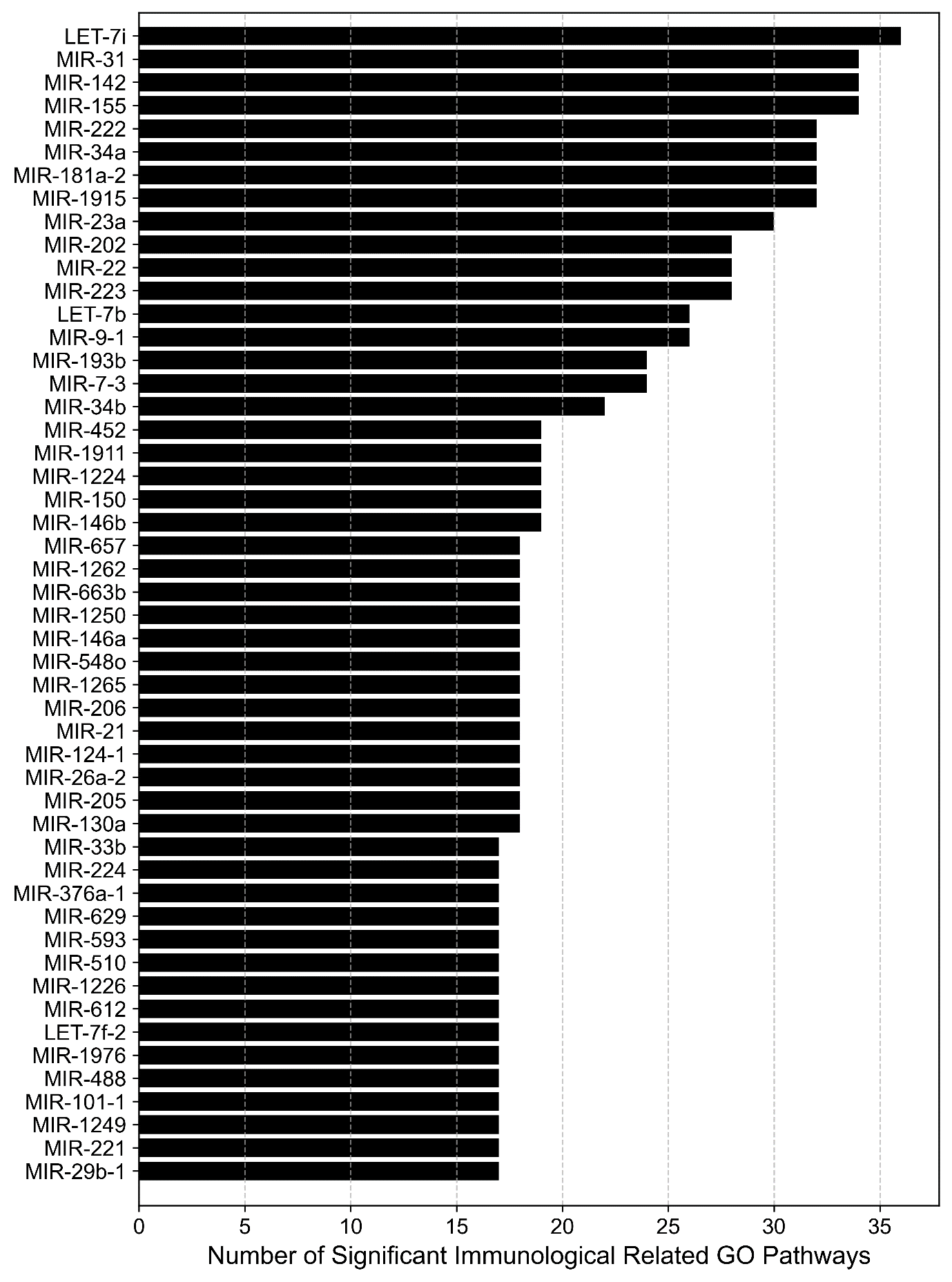
**

**Figure S1: Number of significant immunological related GO pathways miRNAs are correlated with in LinkedOmics platform (HGSC, n=602).** Top 50 miRNAs that have the highest number of significantly correlated immunological related GO pathways are shown. Significant pathways were determined by LinkedOmics GO enrichment analysis (p < 0.05).


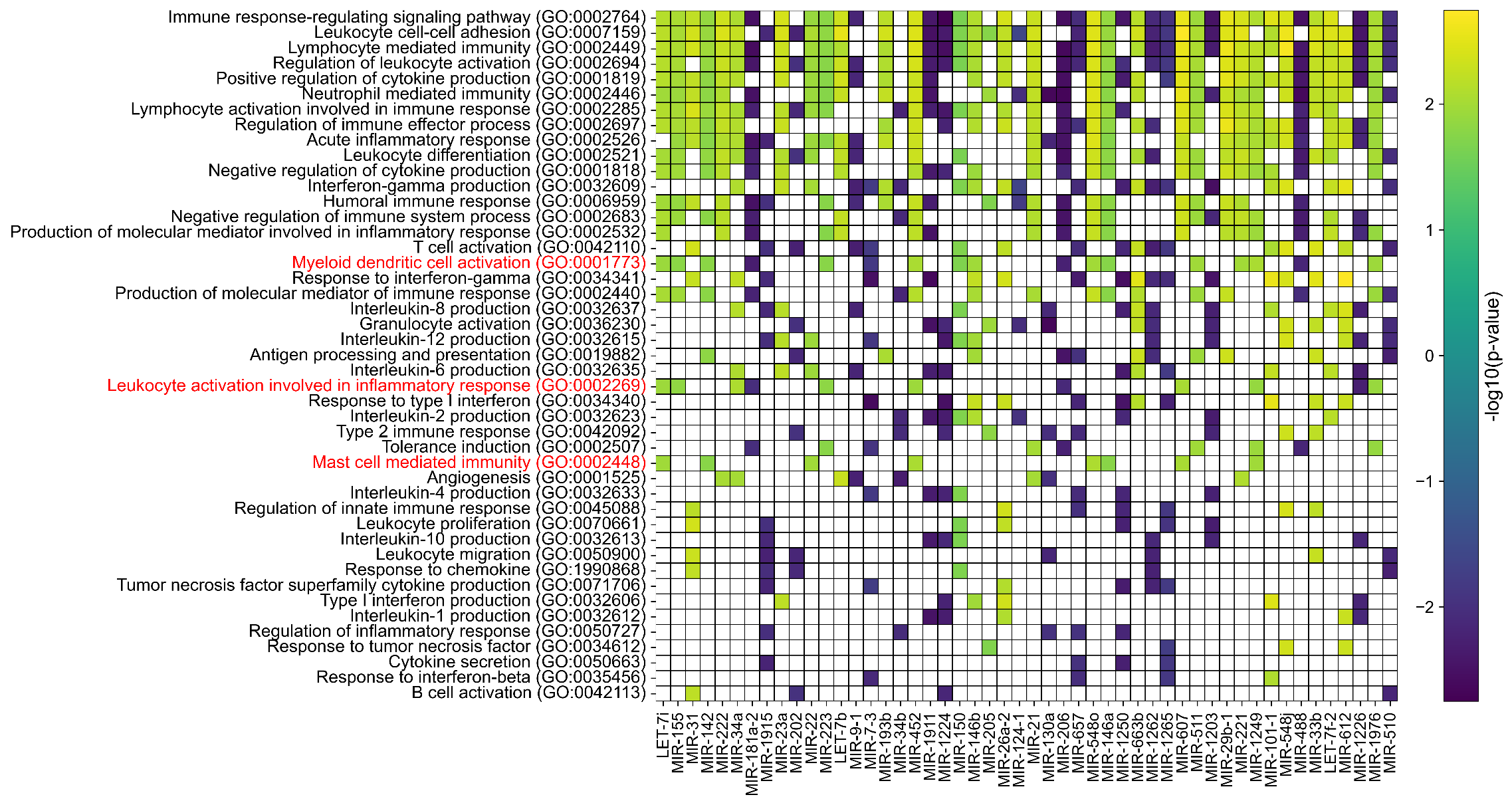


**Figure S2: Correlation bewteen miRNA expression and immunological pathways in HGSC (LinkedOmics platform, n=602).** miRNAs are ranked according to the number of significantly impacted immunological pathways (p < 0.05). The significance of the correlations is indicated by the colour (Log_2_ P-Value).


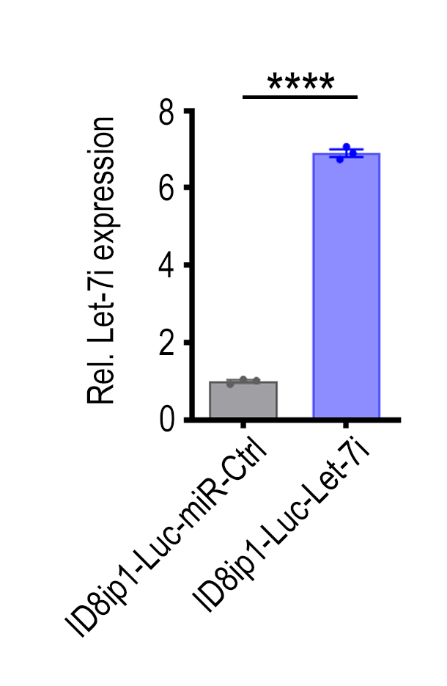


**Figure S3: Let-7i expression in ID8ip1-Luc-miR-Ctrl and ID8ip1-Luc-Let-7i cells.** Relative expression of Let-7i in miR-Ctrl or Let-7i transduced ID8ip1-Luc cells was assessed using qPCR prior to i.p. injection into mice. All bars and error bars represent mean ± SEM (****, p<0.0001, n=3). Statistical analyses were performed by unpaired Student’s *t*-test.

**
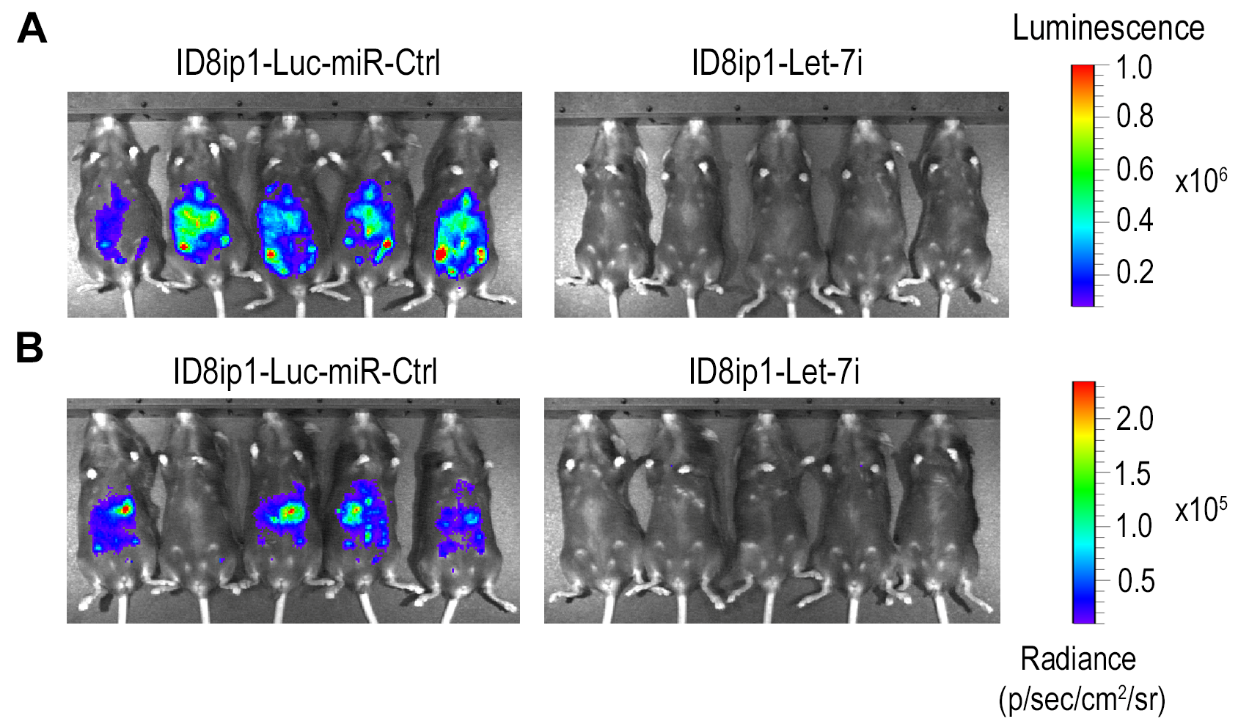
**

**Figure S4: Difference in tumour growth in mice bearing luciferase labelled ID8ip1-Luc-miR-Ctrl cells or ID8ip1-Luc-Let-7i as indicated by luciferase signal**. C57BL/6J mice received i.p. injection with luciferin weekly before experimental end point and representative images from week 4 are shown. The experiment was performed on two separate occasions [(A) and (B)] under the same experimental condition. Bioluminescence signal was captured using the IVIS Lumina X5 imaging system (Combined n=10/treatment group).


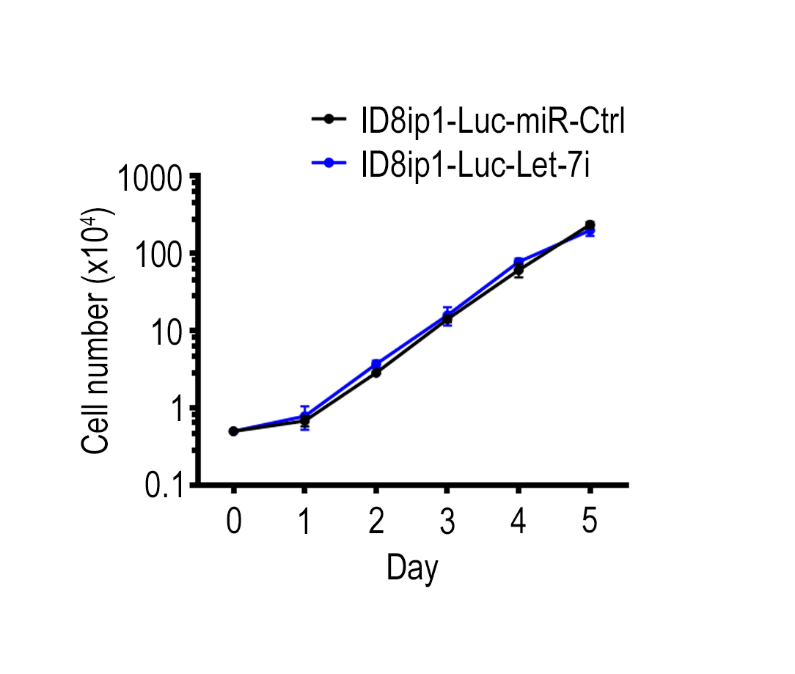


**Figure S5: Impact of constant Let-7i expression on cell growth in ID8ip1-Luc-miR-Ctrl and ID8-ip1-Luc-Let-7i cells *in vitro*.** Cell growth pattern of ID8ip1-Luc-miR-Ctrl and ID8-ip1-Luc-Let-7i cell lines were monitored for 5 days. All bars and error bars represent mean ± SEM (n=6). Statistical analyses were performed by unpaired Student’s *t*-test.


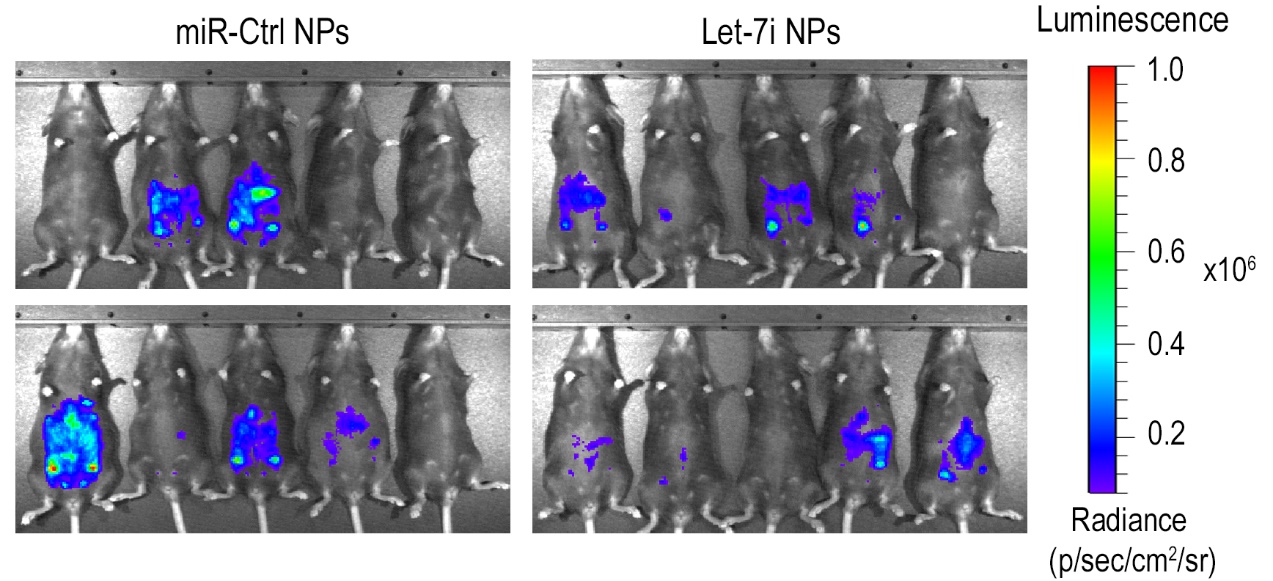


**Figure S6: Difference in tumour growth in mice bearing luciferase labelled ID8ip1-Luc tumours treated with miR-Ctrl or Let-7i NPs as indicated by luciferase signal.** C57BL/6J mice received i.p. injection with luciferin weekly before experimental end point and representative images from week 4 are shown. Ascites development is visible in several mice in the miR-Ctrl NP treatment group which would hinder luciferase signal. Bioluminescence was captured using the IVIS Lumina X5 imaging system (n=10/group).


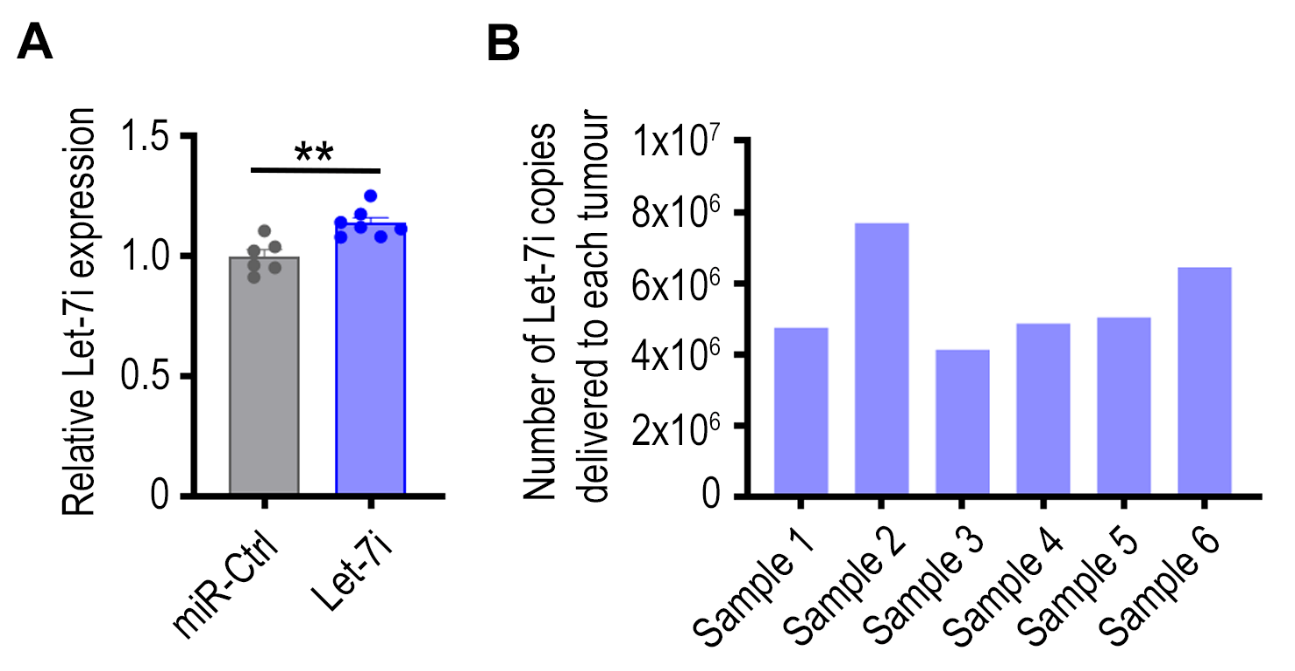


**Figure S7: Quantitation of Let-7i in ID8ip1-Luc tumours following miR-Ctrl or Let-7i NP treatment.** (A) Relative Let-7i expression in ID8ip1-Luc tumours was determined by qPCR in miR-Ctrl or Let-7i NP treatment groups. SNO135 was used as the normalising control for each sample. Bars and error bars represent mean ± SEM (**, p<0.01; miR-Ctrl group, n=6; Let-7i group, n=7, unpaired Student’s *t*-test.). Six and eight tumours were obtained from miR-Ctrl or Let-7i NP treatment at the time of dissection, respectively. Majority of tumours harvested was used for immune profiling assessment with the remaining tissues used for Let-7i quantification. One sample in the Let-7i NP treatment did not have sufficient quantity for RNA extraction for qPCR. (B) Number of Let-7i copies delivered to tumours in the Let-7i NP treatment group, as determined by digital PCR. Number of Let-7i copies detected in the miR-Ctrl NP-treated tumours was considered as background signal and was subtracted out from the number of Let-7i copies detected in the Let-7i NP-treated tumours. RNA quantity was not sufficient for digital PCR analysis for one of the Let-7i-treated tumours. Each bar represents the number of Let-7i copies delivered to tumours in each mouse (n=6).

**
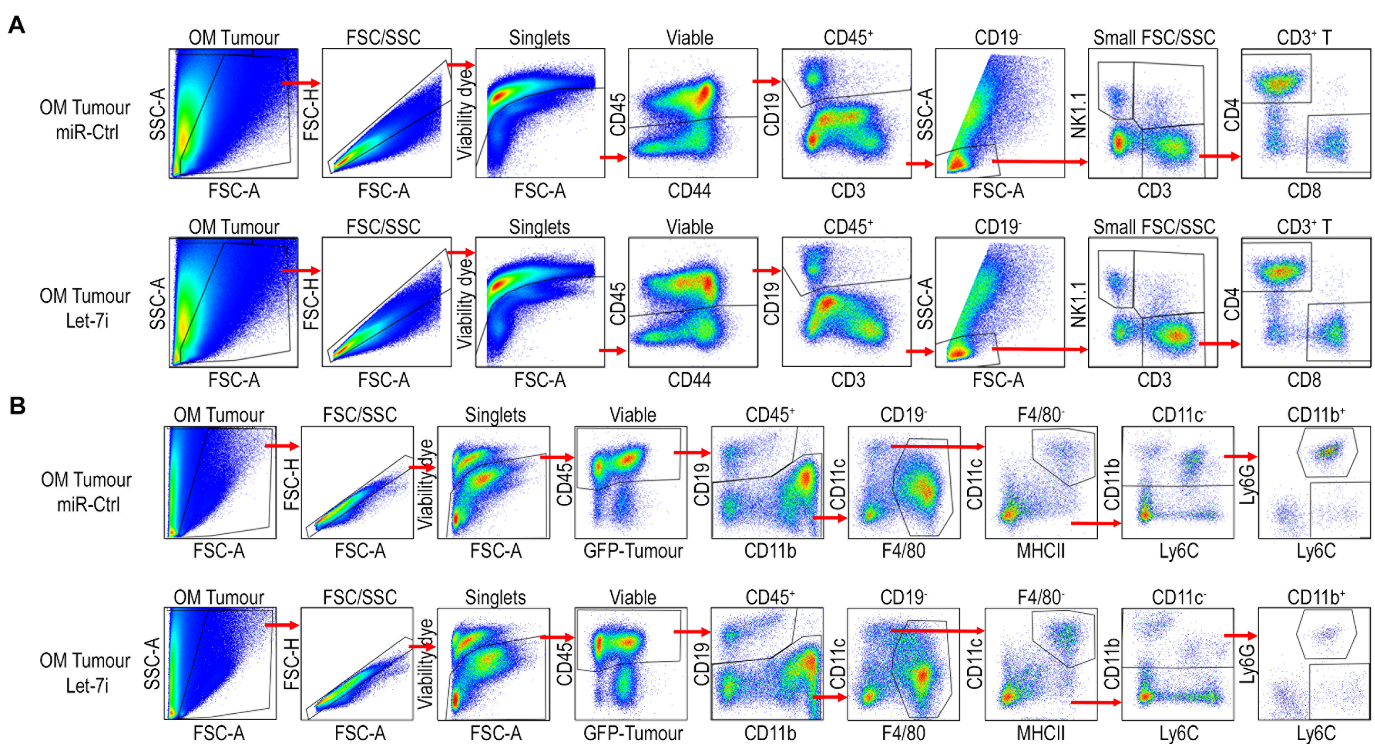
**

**Figure S8: Representative flow plots of lymphoid and myeloid populations in omental tumours following miR-Ctrl and Let-7i NP treatment.** Omental tumours from ID8-ip1 tumour bearing C57BL/6J mice treated with Let-7i or miR-Ctrl NPs were profiled by flow cytometry to examine presence of (A) lymphoid and (B) myeloid immune cell populations. Representative flow images and gating are shown as concatenated samples per group (miR-Ctrl group, n=6; Let-7i group, n=8).


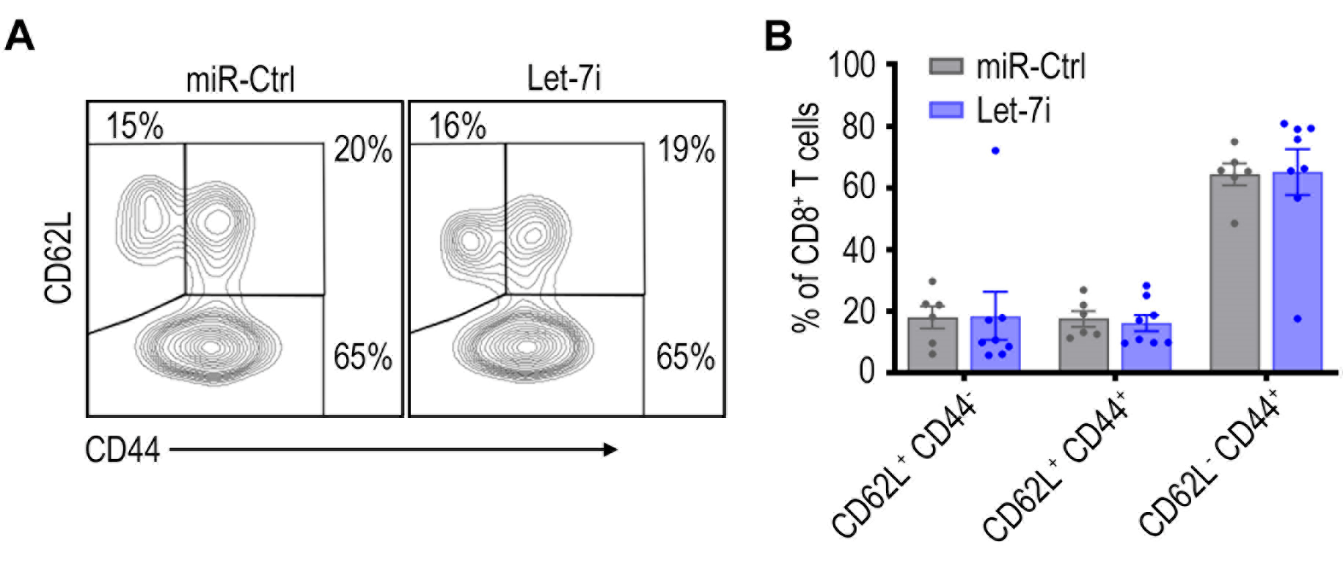
­

**Figure S9: Impact of Let-7i NP treatment on CD8^+^ T cell phenotype in ID8-ip1-Luc tumour model.** ID8-ip1 tumour bearing C57BL/6J mice were treated with Let-7i or miR-Ctrl NPs and the omental tumours were taken at the end point and the phenotype of CD8^+^ T cells were determined by flow cytometry. (A) Representative contour plot (5%) and gating of the concatenated samples from each group and (B) bar graph of the percentage of CD8^+^ T cells as naive (CD62L^+^CD44^-^), central memory (CD62L^+^CD44^+^) and effector/effector memory (CD62L^-^CD44^+^) cells. All bars and error bars represent mean ± SEM (miR-Ctrl group, n=6; Let-7i group, n=8). Statistical analyses were performed by two-way ANOVA with Sidak’s multiple comparisons test.


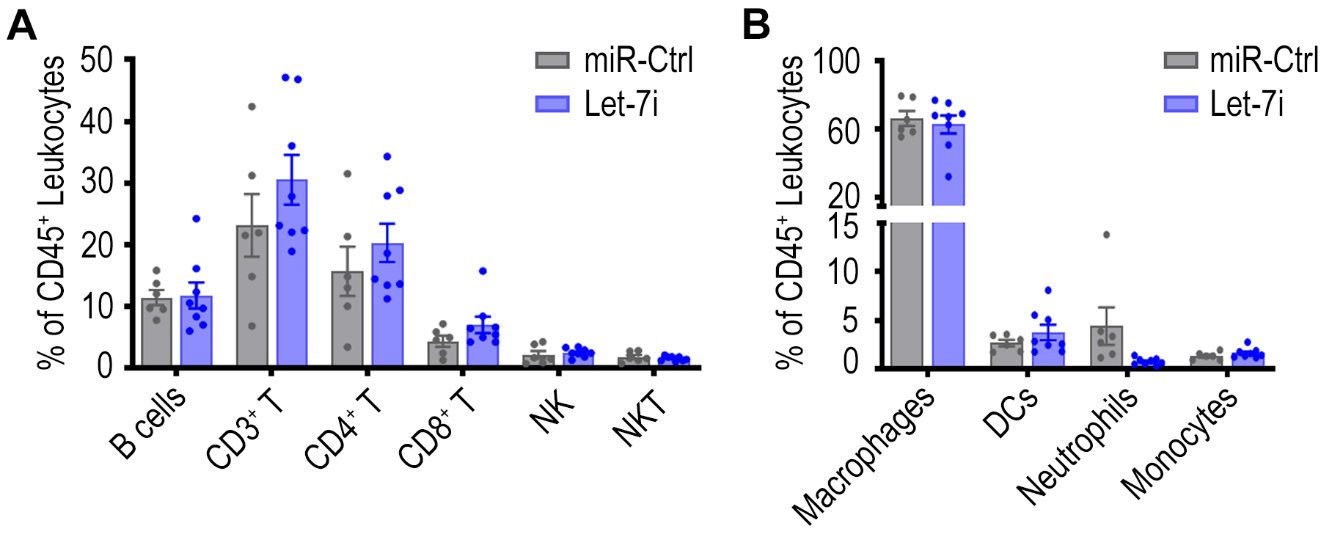


**Figure S10: Impact of Let-7i NP treatment on immune cell populations in ID8-ip1-Luc tumour model.** Omental tumours from ID8-ip1 tumour bearing C57BL/6J mice treated with Let-7i or miR-Ctrl NPs were profiled by flow cytometry to examine the percentage of (A) lymphoid and (B) myeloid immune cells out of all CD45^+^ leukocytes within tumours at experiment endpoint. All bars and error bars represent mean ± SEM (miR-Ctrl group, n=6; Let-7i group, n=8). Statistical analyses were performed by two-way ANOVA with Sidak’s multiple comparisons test.


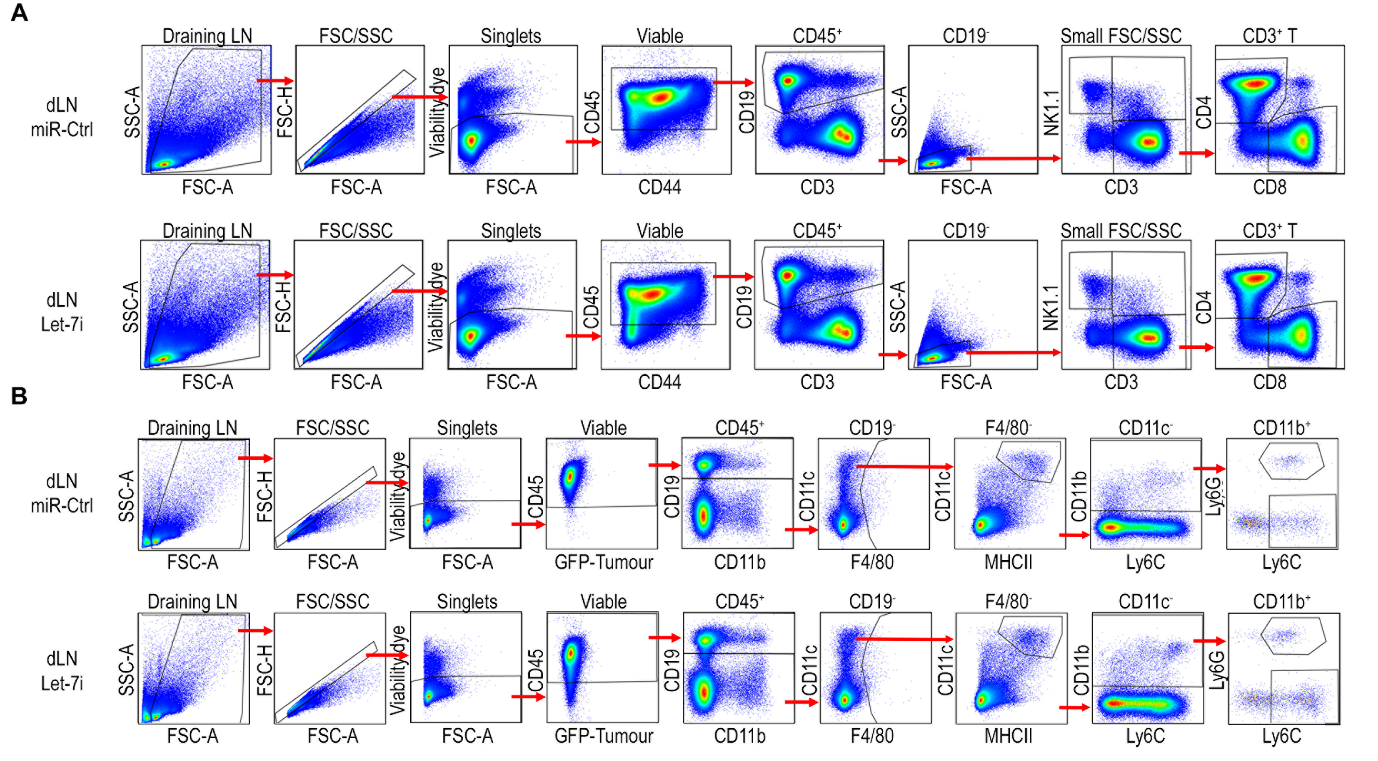


**Figure S11: Representative flow plots of lymphoid and myeloid populations in draining lymph nodes following miR-Ctrl and Let-7i NP treatment.** Inguinal and mesenteric lymph nodes (LNs) from ID8-ip1-Luc tumour bearing mice treated with Let-7i or miR-Ctrl NPs were profiled by flow cytometry to examine the presence of (A) lymphoid and (B) myeloid immune cells. Representative gating and flow images are shown (concatenated samples per group; miR-Ctrl group, n=6; Let-7i group, n=8).


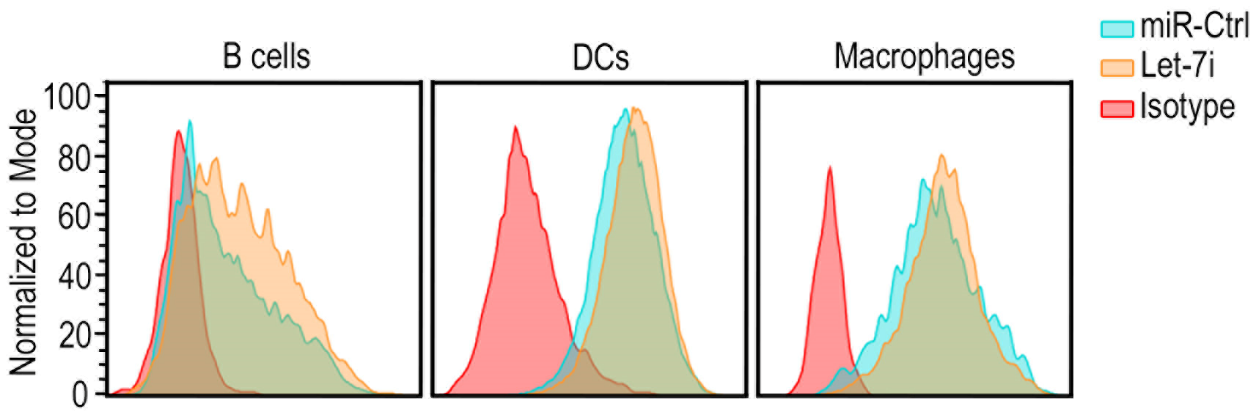


**Figure S12: Impact of Let-7i NP treatment on antigen presenting cell activity in ID8-ip1-Luc tumour model.** Omental tumours from ID8-ip1 tumour bearing C57BL/6J mice treated with Let-7i or miR-Ctrl NPs were profiled by flow cytometry to examine the CD86, a surrogate marker for activity, Mean Fluorescence Intensity (MFI) on antigen presenting cells (APCs; B cells, DCs, and macrophages) within tumours at experiment endpoint. Representative histograms (concatenated samples per group) are shown (miR-Ctrl group, n=6; Let-7i group, n=8).

**Supplementary Table 1:** Antibodies used for flow cytometry analyses.

| **Antigen** | **Fluorophore** | **Dilution** | **Company** | **Catalogue no.** |
| --- | --- | --- | --- | --- |
| L/D Ghost Dye | BV510 | 1/800 | Tonbo | 13-0870-T500 |
| CD11b | PerCP-Cy5.5 | 1/500 | BioLegend | 101228 |
| CD11c | APC | 1/300 | Biolegend | 117310 |
| CD19 | BV605 | 1/300 | BioLegend | 115540 |
| CD3 | PE-Cy7 | 1/200 | BioLegend | 100220 |
| CD4 | APC-Cy7 | 1/300 | BioLegend | 100414 |
| CD44 | FITC | 1/200 | Biolegend | 103022 |
| CD62L | AF700 | 1/300 | ThermoFisher | 56-0621-82 |
| CD8a | BV785 | 1/300 | BD Biosciences | 563332 |
| CD45.2 | BV650 | 1/200 | Biolegend | 109836 |
| CD86 | PE | 1/200 | Ebioscience | 12-0862-83 |
| F4/80 | PE-Cy7 | 1/300 | BioLegend | 123114 |
| I-A/I-E (MHC II) | Pacific Blue | 1/500 | BioLegend | 107620 |
| Ly6G | AF700 | 1/250 | BioLegend | 127622 |
| Ly6C | APC-e780 | 1/400 | eBiosciences | 47-5932-80 |
| NK1.1 | PerCP-Cy5.5 | 1/300 | eBiosciences | 45-5941-82 |
| Rat IgG2a, κ | PE | 1/200 | Thermo-Fisher | 12-4321-80 |

**Supplementary Table 2:** Markers used to define distinct immune cell populations.

| **Immune cell population** | **Markers** |
| --- | --- |
| Dendritic cells (DCs) | Ghost dye^-^, CD45^+^, CD19^-^, CD11c^+^, MHC-II^+^ |
| Macrophages | Ghost dye^-^, CD45^+^, CD19^-^, F4/80^+^, CD11b^+^ |
| Neutrophils | Ghost dye^-^, CD45^+^, CD19^-^, exclude macrophages (F4/80^+^ CD11b^+^), exclude DCs (CD11c^+^ MHC-II^+^), CD11b^+^, Ly6G^+^, Ly6C^int^ |
| Monocytes | Ghost dye^-^, CD45^+^, CD19^-^, exclude macrophages (F4/80^+^ CD11b^+^), exclude DCs (CD11c^+^ MHC-II^+^), CD11b^+^, Ly6G^-^, Ly6C^+^ |
| B cells | CD45^+^, CD19^+^, CD3^-^, CD11b^-^, CD11c^-^, NK1.1^-^ |
| Natural Killer (NK) cells | CD45^+^, CD19^-^, CD3^-^, CD11b^-^, CD11c^-^, NK1.1^+^ |
| Natural Killer T (NKT) cells | CD45^+^, CD19^-^, CD3^+^, CD11b^-^, CD11c^-^, NK1.1^+^ |
| CD3^+^ T cells | CD45^+^, CD19^-^, CD3^+^, CD11b^-^, CD11c^-^, NK1.1^-^ |
| CD4^+^ T cells | CD45^+^, CD19^-^, CD3^+^, CD4^+^, CD8^-^, CD11b^-^, CD11c^-^, NK1.1^-^ |
| CD8^+^ T cells | CD45^+^, CD19^-^, CD3^+^, CD4^-^, CD8^+^, CD11b^-^, CD11c^-^, NK1.1^-^ |

**Supplementary Table 3:** Categorisation of GO Terms.

| **Cytokine Production** | **Immune Response Activation** | **Immune Response Regulation** | **Inflammatory Response** | **Cellular Immunity** | **Cellular Differentiation** | **Response to Cytokines** |
| --- | --- | --- | --- | --- | --- | --- |
| Interleukin-1 production (GO:0032612) | Lymphocyte activation involved in immune response (GO:0002285) | Regulation of response to cytokine stimulus (GO:0060759) | Leukocyte activation involved in inflammatory response (GO:0002269) | Neutrophil mediated immunity (GO:0002446) | Hematopoietic progenitor cell differentiation (GO:0002244) | Response to type I interferon (GO:0034340) |
| Interleukin-10 production (GO:0032613) | Angiogenesis (GO:0001525) | Negative regulation of cytokine production (GO:0001818) | Inflammatory response to antigenic stimulus (GO:0002437) | Mast cell mediated immunity (GO:0002448) | Leukocyte differentiation (GO:0002521) | Response to interferon-gamma (GO:0034341) |
| Interleukin-12 production (GO:0032615) | Myeloid dendritic cell activation (GO:0001773) | Positive regulation of cytokine production (GO:0001819) | Antigen processing and presentation (GO:0019882) | Lymphocyte mediated immunity (GO:0002449) | Myeloid cell differentiation (GO:0030099) | Response to tumor necrosis factor (GO:0034612) |
| Interleukin-13 production (GO:0032616) | T cell activation (GO:0042110) | Tolerance induction (GO:0002507) | Acute inflammatory response (GO:0002526) | Granulocyte activation (GO:0036230) | Cellular response to vascular endothelial growth factor stimulus (GO:0035924) | Response to interferon-alpha (GO:0035455) |
| Interleukin-17 production (GO:0032620) | B cell activation (GO:0042113) | Negative regulation of immune system process (GO:0002683) | Production of molecular mediator involved in inflammatory response (GO:0002532) |  | Asymmetric cell division (GO:0008356) | Response to interferon-beta (GO:0035456) |
| Interleukin-2 production (GO:0032623) | Macrophage activation (GO:0042116) | Regulation of leukocyte activation (GO:0002694) | Neuroinflammatory response (GO:0150076) |  | Leukocyte cell-cell adhesion (GO:0007159) | Response to interleukin-1 (GO:0070555) |
| Interleukin-4 production (GO:0032633) | Natural killer cell activation (GO:0030101) | Regulation of immune effector process (GO:0002697) | Organ or tissue specific immune response (GO:0002251) |  | Leukocyte migration (GO:0050900) | Response to interleukin-4 (GO:0070670) |
| Interleukin-5 production (GO:0032634) | Mast cell activation (GO:0045576) | Immune response-regulating signaling pathway (GO:0002764) | Vascular endothelial growth factor production (GO:0010573) |  | Leukocyte proliferation (GO:0070661) | Response to interleukin-6 (GO:0070741) |
| Interleukin-6 production (GO:0032635) | Type 2 immune response (GO:0042092) | Regulation of inflammatory response (GO:0050727) | Vascular endothelial growth factor receptor signaling pathway (GO:0048010) |  |  | Response to transforming growth factor beta (GO:0071559) |
| Interleukin-8 production (GO:0032637) | Somatic diversification of immune receptors (GO:0002200) | Regulation of innate immune response (GO:0045088) | Spleen development (GO:0048536) |  |  | Response to interleukin-7 (GO:0098760) |
| Type I interferon production (GO:0032606) | Humoral immune response (GO:0006959) |  | Regulation of hemopoiesis (GO:1903706) |  |  | Response to interleukin-12 (GO:0070671) |
| Interferon-gamma production (GO:0032609) |  |  |  |  |  | Response to leukemia inhibitory factor (GO:1990823) |
| Tumor necrosis factor superfamily cytokine production (GO:0071706) |  |  |  |  |  | Response to chemokine (GO:1990868) |
| Production of molecular mediator of immune response (GO:0002440) |  |  |  |  |  | Leukocyte apoptotic process (GO:0071887) |
| Chemokine production (GO:0032602) |  |  |  |  |  | Inflammatory cell apoptotic process (GO:0006925) |
| Cytokine secretion (GO:0050663) |  |  |  |  |  | Myeloid cell apoptotic process (GO:0033028) |
|  |  |  |  |  |  | Anoikis (GO:0043276) |
